# A case of humidifier lung with a difficult differential diagnosis from COVID-19

**DOI:** 10.1093/omcr/omab100

**Published:** 2021-10-26

**Authors:** Mariko Ono, Yoko Nagatomo, Hayato Kinoshita, Yukihisa Takeda, Hiroyuki Nakamura, Kazutetsu Aoshiba

**Affiliations:** 1 Department of Respiratory Medicine, Tokyo Medical University Ibaraki Medical Center, Ibaraki, Japan; 2 Department of Respiratory Medicine, Tokyo Medical University, Tokyo, Japan

## Abstract

Acute respiratory illnesses that presented with diffuse ground-glass opacities (GGOs) on chest computed tomography (CT) scan suggest the diagnosis of coronavirus disease 2019 (COVID-19). However, many other diseases show similar CT findings, which often offer a difficult differential diagnosis. Here, we report a case of humidifier lung, a rare phenotype of hypersensitivity pneumonitis (HP), which mimicked COVID-19. A 71-year-old man was admitted because of dyspnea and diffuse GGOs found on chest CT scan. Although COVID-19 was initially suspected, his symptoms rapidly improved by the next day. A medical interview revealed that he had started using an ultrasonic humidifier 1 month ago. A high-resolution CT (HRCT) scan showed ill-defined centrilobular nodules and mosaic attenuation, which are typical of HP but atypical of COVID-19. The inhalation challenge test confirmed the diagnosis of humidifier lung. History-taking of humidifier use and a precise HRCT interpretation are helpful to differentiate it from COVID-19.

## INTRODUCTION

In the current coronavirus disease 2019 (COVID-19) pandemic, acute respiratory illnesses presenting with diffuse or multifocal ground-glass opacities (GGOs) on chest computed tomography (CT) scan suggest the diagnosis of severe acute respiratory syndrome coronavirus 2 (SARS-CoV-2) infection [1, 2]. However, many other diseases show CT findings similar to those of COVID-19, which often offer a difficult differential diagnosis. The disease entities mimicking COVID-19 include hypersensitivity pneumonitis (HP), which is caused by Types III and IV allergic reactions acquired through repeated inhalation of antigens [3]. Here, we report a case of a patient with HP caused by ultrasonic humidifier (humidifier lung), who presented with cough, dyspnea and diffuse GGO shadows on chest CT scan. The patient was initially suspected of having COVID-19, but he was finally diagnosed with humidifier lung based on the history of humidifier use and interpretation of centrilobular lesions found on the high-resolution CT (HRCT) scan, a combination of which was useful in differentiating it from COVID-19.

## CASE REPORT

**Figure 1:**
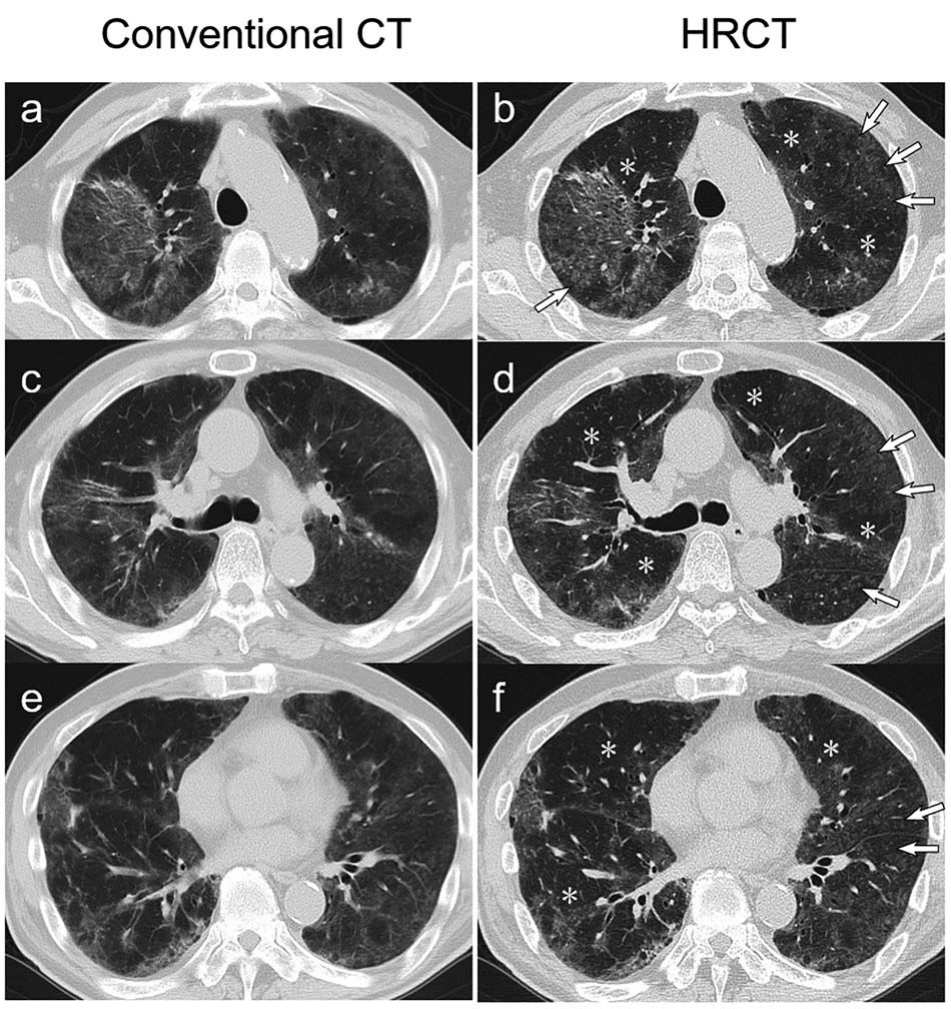
Chest CT scan images taken during hospitalization. A conventional 5-mm-thick CT scan (**a**, **c** and **e**) and high-resolution 2-mm-thick CT (HRCT) scan (**b**, **d** and **f**) taken at the levels of the aortic arch (a and b), trachea carina (c and d) and aortic valve (e and f) show diffuse ground-glass opacities (GGOs) with relatively predominant peripheral distribution on the bilateral lungs. The HRCT scan shows ill-defined centrilobular nodules (*arrows*) and mosaic attenuation (*asterisks*), which are characteristics of hypersensitivity pneumonitis.

**Figure 2:**
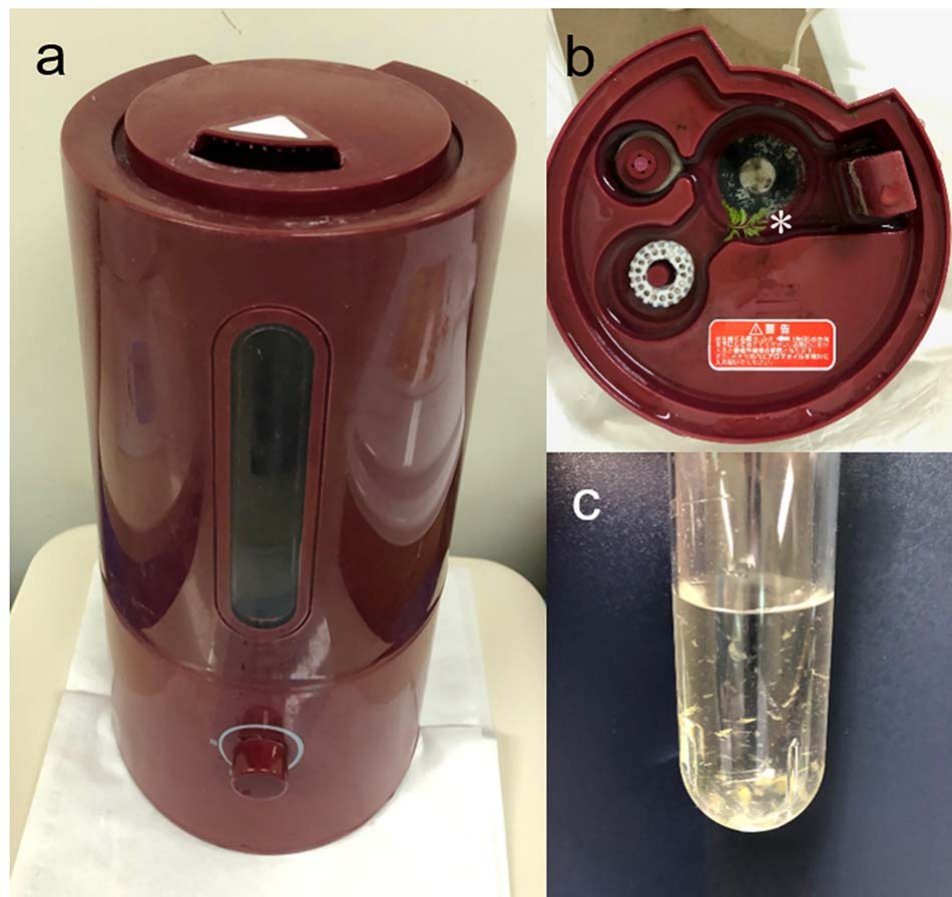
The ultrasonic humidifier used by the patient. (**a**) The humidifier. (**b**) The inside of the humidifier tank, which was not cleaned. The *asterisk* indicates some leaves that fell into the tank from a plant located next to the humidifier. (**c**) Turbid humidifier tank water containing dirty suspended materials.

**Figure 3:**
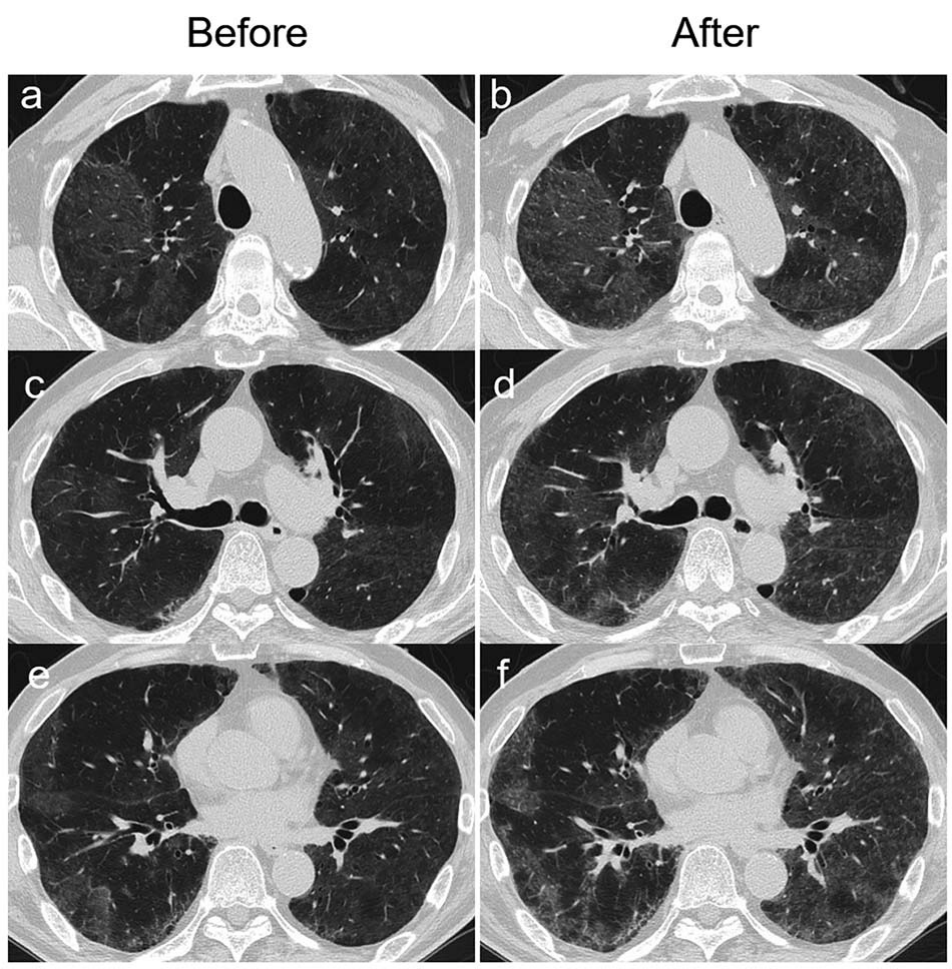
Chest HRCT images taken before and 9 h after the inhalation challenge test (ICT) with humidifier water performed on hospitalization Day 9. The HRCT scan taken before the ICT (**a**, **c** and **e**) shows the improvement of GGOs compared to the HRCT scan taken on the hospitalization Day 1 (Fig. 1b, d and f). After the ICT, GGOs worsened again (**b**, **d** and **f**).

A 71-year-old Japanese man was referred to our hospital with a 1-week history of cough and dyspnea. He had a history of cigarette smoking (36 pack-years) but denied having any pets or recent sick contacts. He had tachypnea (26 breaths/min) and hypoxemia (SpO2, 93% on room air), with no fever, wheezes, lung crackles and abnormal heart sounds. Laboratory blood tests revealed a leukocytosis and increased levels of C-reactive protein (CRP) and Krebs von den Lunges-6 (KL-6; Table 1). A chest X-ray and conventional CT scan taken at an emergency department showed diffuse GGOs with relatively predominant peripheral distribution in bilateral lung fields (Fig. 1). The SARS-CoV-2 antigen and SARS-CoV-2 PCR tests were negative. However, COVID-19 could not be ruled out, and the patient was admitted to an isolation ward in a hospital. Empiric antibiotics (ampicillin/sulbactam and azithromycin) were started, but the results of blood culture and urinary pneumococcal and *Legionella* antigen tests were negative. His symptoms rapidly improved by the next day, and his chest X-ray shadows were almost resolved after 3 days of hospitalization. The white blood cell counts and CRP levels were normalized by Day 5. After the second negative PCR test for SARS-CoV-2, the patient was moved to a general ward. Based on the rapid improvement after hospitalization, HP was suspected. A medical interview revealed that the patient had been using an ultrasonic humidifier during winter and started to use it 1 month before admission, without changing the water or cleaning the tank (Fig. 2). The HRCT images reconstructed after hospitalization identified ill-defined centrilobular nodules and mosaic attenuation superimposed with GGOs, which are HRCT features typical of HP but atypical of COVID-19 (Fig. 1) [1, 2, 4]. On hospitalization Day 9, an inhalational challenge test (ICT) was performed with the humidifier. Six hours after the test, the patient developed cough, dyspnea, fever (37.5°C) and hypoxemia with increased alveolar-arterial oxygen tension difference (53 mmHg at room air). Pulmonary function tests performed before and 6 h after the ICT revealed post-inhalation reductions in forced vital capacity from 2.95 l (75.6% of predicted) to 2.22 l (56.9% of predicted). A HRCT scan performed 9 h after the ICT revealed the re-aggravation of GGOs (Fig. 3). Blood tests performed on the next day showed an increased white blood cell count (33 000/μl) and CRP level (9.61 mg/dl). Thus, humidifier lung was diagnosed. Various microorganisms, such as *Stenotrophomonas maltophilia*, *Acinetobacter baylyi* and *Cladosporium sp.* were isolated from the humidifier tank water. After the ICT, the patient was started on prednisolone therapy (30 mg/day; 0.5 mg/kg/day), which was reduced and tapered off thereafter. The patient was discharged without any symptoms after 16 days of hospitalization.

**Table 1:** Laboratory data of the patient

Test	Results	Units	Reference range
**Hematology**			
White blood cells	17.3	×10^3^/μl	4.0–8.5
Neutrophil	81.9	%	
Lymphocyte	14.1	%	
Basophil	0.3	%	
Eosinophil	1.1	%	
Monocyte	2.6	%	
Red blood cells	4.4	×10^3^/μl	4.0–5.5
Hemoglobin	12.8	g/dl	13–18
Hematocrit	39.0	%	36–52
Platelets	622	×10^3^/μl	160–400
**Chemistry**			
LDH	169	U/l	124–222
KL-6	1300	U/ml	< 500
SP-D	86.5	ng/ml	< 110
CRP	4.4	mg/dl	0.0–0.3
Anti-*Trichosporon asahii* antibodies	Negative		

*Abbreviations:* LDH, lactate dehydrogenase; KL-6, Krebs von den Lungen-6; SP-D, surfactant protein-D; CRP, C-reactive protein.

## DISCUSSION

Humidifier lung is a rare phenotype of HP caused by the inhalation of humidifier vapors contaminated with bacteria, fungi or endotoxins [5, 6]. It often presents with acute respiratory symptoms, such as fever, cough and dyspnea, and chest CT images showing diffuse or multifocal GGOs, which often require differentiation from COVID-19.

The typical CT features of COVID-19 include peripheral, bilateral GGOs with or without consolidations [1, 2], which complement the diagnosis of COVID-19. On the other hand, the presence of centrilobular nodules suggests an alternative diagnosis, such as HP, including humidifier lung [1]. The current international guidelines for the diagnosis of HP recommend the use of HRCT, which allows the identification of typical features of HP, as seen in our case, which had GGOs mixed with ill-defined centrilobular nodules reflecting granulomatous bronchiolitis and mosaic attenuation reflecting airway trapping [4]. It should be noted that the clinical characteristics of humidifier lung and other HP phenotypes are somewhat different [5–7]. For example, compared with patients with summer-type HP, which is the most common HP phenotype in Japan, patients with humidifier lung have faster disease progression, lower Krebs von den Lunges-6 (KL-6) levels, fewer centrilobular nodules, more frequent subpleural consolidation, higher neutrophil but lower lymphocyte numbers in bronchoalveolar lavage fluids and lower prevalence of granuloma on histological examination [5–7]. These differences may be due to inflammation induced by the inhalation of humidifier vapors contaminated with endotoxins [5, 8]. In fact, the CT findings in our patient showed a higher density area compared with the routine density of GGOs in the peripheral and right upper lung (Fig. 1). These slightly atypical findings for HP may make it more difficult to differentiate between COVID-19 and humidifier lung.

Since the COVID-19 outbreak, many people in Japan started using humidifiers under the influence of COVID-19 prevention advertisements on television and the internet [9]. Interestingly, a single-center hospital study has shown an increase in Japanese patients with humidifier lung during the COVID-19 pandemic [9]. However, humidifier lung is still rare, not well-recognized among physicians, and may be confused with COVID-19. It can be improved by avoiding the use of humidifiers. Since PCR for SARS-CoV-2 have been reported to be ~70% [10], some false-negatives results may occur. Therefore, differentiation of humidifier lung from COVID-19 requires good history-taking and a precise interpretation of centrilobular changes on HRCT [11]. The latter are not typically seen in patients with COVID-19.

## CONFLICT OF INTEREST

The authors have no conflicts of interest to disclose.

## FUNDING

No sources of funding were used.

## ETHICAL APPROVAL

This case report meets the standards of Tokyo Medical University ethical committee. All personal identifiers were removed from the manuscript.

## CONSENT

Written consent from the patient was obtained for submission and publication of the case details and images.

## GUARANTOR

The nominated guarantor is KA.
